# Anticancer Potential of Antimicrobial Peptides: Focus on Buforins

**DOI:** 10.3390/polym16060728

**Published:** 2024-03-07

**Authors:** Ana Maria Tolos (Vasii), Cristian Moisa, Mihaela Dochia, Carmen Popa, Lucian Copolovici, Dana Maria Copolovici

**Affiliations:** 1 Institute for Research, Development and Innovation in Technical and Natural Sciences, Aurel Vlaicu University, Elena Dragoi St., Nr. 2, 310330 Arad, Romania; anamaria.tolos@yahoo.com (A.M.T.); cristian.moisa@uav.ro (C.M.); dochiamihaela@yahoo.com (M.D.); camy_popa2007@yahoo.com (C.P.); lucian.copolovici@uav.ro (L.C.); 2 Biomedical Sciences Doctoral School, University of Oradea, University St., Nr. 1, 410087 Oradea, Romania; 3 Faculty of Food Engineering, Tourism and Environmental Protection, Aurel Vlaicu University, Elena Dragoi St., Nr. 2, 310330 Arad, Romania

**Keywords:** antimicrobial peptides, anticancer, buforins

## Abstract

In seeking alternative cancer treatments, antimicrobial peptides (AMPs), sourced from various life forms, emerge as promising contenders. These endogenous peptides, also known as host defense peptides (HDPs), play crucial roles in immune defenses against infections and exhibit potential in combating cancers. With their diverse defensive functions, plant-derived AMPs, such as thionins and defensins, offer a rich repertoire of antimicrobial properties. Insects, amphibians, and animals contribute unique AMPs like cecropins, temporins, and cathelicidins, showcasing broad-spectrum activities against bacteria, fungi, and viruses. Understanding these natural peptides holds significant potential for developing effective and targeted therapies against cancer and infectious diseases. Antimicrobial peptides (AMPs) exhibit diverse structural characteristics, including α-helical, β-sheet, extended, and loop peptides. Environmental conditions influence their structure, connecting to changes in cell membrane hydrophobicity. AMPs’ actions involve direct killing and immune regulation, with additional activities like membrane depolarization. In this review, we focus on antimicrobial peptides that act as anticancer agents and AMPs that exhibit mechanisms akin to antimicrobial activity. Buforin AMPs, particularly Buforin I and II, derived from histone H2A, demonstrate antibacterial and anticancer potential. Buforin IIb and its analogs show promise, with selectivity for cancer cells. Despite the challenges, AMPs offer a unique approach to combat microbial resistance and potential cancer treatment. In various cancer types, including HeLa, breast, lung, ovarian, prostate, and liver cancers, buforins demonstrate inhibitory effects and apoptosis induction. To address limitations like stability and bioavailability, researchers explore buforin-containing bioconjugates, covalently linked with nanoparticles or liposomes. Bioconjugation enhances specificity-controlled release and combats drug resistance, presenting a promising avenue for targeted cancer treatment. Clinical translation awaits further evaluation through in vivo studies and future clinical trials.

## 1. Introduction

Non-communicable illnesses are becoming a widespread global public health issue. Cancer is the second leading cause of morbidity and mortality worldwide [1]. It continues to be a major health burden, estimated to have killed 10 million people in 2018 and up to 12 million in 2020, despite considerable research and decades of therapeutic approaches [2,3]. Most antitumor medications used in cancer treatment cannot distinguish between cancer cells and normal cells (they are not selective), causing side effects such as hair loss, diarrhea, and a weakened immune system. Acquired drug resistance, which, according to the literature, accounts for up to 90% of cancer patients’ mortality, is one of the main challenges of chemotherapy [4]. Consequently, scientists in the pharmaceutical industry have been attempting to create chemotherapy medications that are less hazardous to healthy cells and have more efficacy [5]. The primary goal of the researcher is to develop new medicines that target cancers while also extending survival times, reducing unfavorable side effects, and improving patient quality of life [6].

Several approaches have been studied to reduce the negative effects of chemotherapy. Two of these have received particular notice: encapsulation in nanocarriers and conjugation of drug molecules to water-soluble polymers [7]. There is a significant possibility of improving various approaches to treating cancer thanks to the swift development of nanotechnology and the discovery of nanomedical agents [8].

Creating antibody–drug conjugates (ADCs), which combine the cytotoxic effects of chemotherapy with monoclonal antibodies, has demonstrated promising efficacy with minimal side effects compared to conventional treatment [9]. Recently, interest has grown in nanotheranostics, a theory that integrates many capabilities into a single nanotechnology-based, multifunctional system for drug delivery and cancer imaging in one package [10]. Often made from peptides, antibodies, integrin receptor ligands, aptamers, and other molecular components, these medications consist of recognition domains (detect appropriate markers on endothelial cells) and effector domains (contribute to the curative effect). Due to their small size, precise chemical structure, and stability, peptides utilized as ligands offer significant benefits in cellular targeting. The large-scale, high-purity production of peptides is simple and economical. The peptides are highly selective to target cells and tissues, biocompatible, and can simultaneously have many ligands coupled to the delivery vehicle to maximize binding to the target [7]. Because of their target particularity to corresponding endogenous receptors, a variety of natural peptide analogs, including hormone peptides, somatostatin, cholecystokinin/gastrin, bombesin, and Arg-Gly-Asp (RGD-receptor-mediated peptide analogs), receptor-targeting peptides, dual-receptor-targeting peptides, head-to-tail cyclic peptides, peptide vaccines, theranostic agents, and self-assembled peptide-based nanoprobes have been proposed for diagnosis and treatment. Their applications as therapeutic agents are constrained by proteolytic degradation. There are several ways to avoid this inconvenience, including cyclization, hybridization, the addition of D-amino acids, and alteration of the peptide’s C- and N-termini by therapeutic or targeted compounds [6].

A new possible anticancer treatment is using selenium-conjugated peptides (STPs). Selenium compounds can potentially slow cancer cell development and trigger apoptosis in cancer cell culture models [11]. Selenobevacizumab and Selenotrastuzumab were developed by Khandelwal et al. by combining the humanized IgG1 antibodies with redox selenium and assessed their efficacy against the normal human mammary epithelial cell line and the triple-negative breast cancer (TNBC) cell lines [12]. Also, the antitumor action of Seleno-β- lactoglobulin was analyzed by Yu et al. on human gastric cancer cells [13]. Zeng et al. combined the RGD peptide with the selenadiazole derivative (RGD-SeD) for better selectivity and specificity. RGD-SeD showed antitumor effectiveness in αVβ3 integrin-overexpressing HepG2 liver cancer cells [14]. According to Yang et al., the selenium-conjugated selenadiazole peptide could enter cells by receptor-mediated endocytosis via clathrin-mediated and nystatin-dependent lipid-raft-mediated pathways after being attached to nanoparticles. Ranjitha et al. observed a comparable impact on colorectal cancer cells [15].

Other promising anticancer therapeutic compounds are marine peptides. These have gained attention for a variety of reasons. Unlike proteins or antibodies, they are smaller, easier to manufacture, more easily changed, capable of traversing cell membranes, less likely to interact with other drugs, and more versatile in chemical and biological processes. Another benefit is fewer adverse side effects from liver or renal buildup [16].

For the treatment of diabetes, cardiovascular diseases, and several cancers, including head and neck, brain, breast, prostate, cervical, and ovarian cancers, peptide drugs have been created as prospective targeted treatments. Clinical trials for more than 60 therapeutic peptide medications are currently being conducted. The U.S. Food and Drug Administration (FDA) has approved 15 peptides in the last few years [17]. These peptide drugs serve as radionuclide carriers (b, g-emitters) and inhibitors of angiogenesis, cytotoxic medicines, and fluorescence probes (visible, Near-IR, Far-IR). The peptide analogs can also be utilized as hormones and vaccines [6].

This review aimed to present the identification and applications of AMPs with anticancer potential, with a main candidate being buforin and its derivatives.

## 2. Antimicrobial Peptides (AMPs) with Anticancer Potential

The use of traditional chemotherapies for cancer treatment, which are based on hormone agonists/antagonists, antimetabolites, and alkylating drugs, is restricted due to the development of multi-drug resistance by cancer cells and significant side effects [18]. A number of bodily functions are also impacted by these medications, which might result in unfavorable side effects include anemia, exhaustion, constipation, diarrhea, chest discomfort, mucositis, rash, and vomiting [19]. A suitable and focused treatment plan may be suggested in light of the difficulties in cancer therapy, with the potential to enhance quality of life and results.

As an alternative to existing chemotherapeutic treatments, antimicrobial peptides (AMPs) with cancer-selective cytotoxicity, have drawn attention recently. Compared to the anticancer therapies currently in use, these peptides have a number of advantages, including reduced intrinsic cytotoxicity, a lower chance of resistance developing, and additive effects in combination therapy [18,20].

AMPs offer many benefits, such as rapid onset of action, low toxicity, low risk of resistance, and broad-spectrum activity, which have made them attractive candidates for clinical treatments. The clinical application of AMPs as antineoplastic drugs is hampered by a number of factors, including their high cost of manufacture, short half-life, severe immune reaction, and collateral damage on normal mammalian cells, despite the fact that there is promise for their translation into clinical practice [21].

A presentation of the list of several AMPs with anticancer activities, like lactoferricin, magainin II, NRC-3, NRC-7, BR2, pardaxin, cecropin B, gomesin, LL-37, buforin IIb, CopA3, gaegurins, citropin 1.1, tachyplesin I-III, their mechanism of actions, and the sequence-based computational methods that are free and suitable to discover new peptide sequences with potential anticancer properties was published in a review by Kordi et al. [22].

Lactoferricin-derived peptides both from bovine and human milk were shown to present in vitro anticancer activity toward leukemia and breast cancer cells when the antimicrobial core sequence is maintained [23].

Cecropins are found in the skin of various frog species, including the African clawed frog and the leopard frog *Rana pipiens.* They have broad-spectrum activity against bacteria, viruses, and fungi [24], and also against ovarian and endometrial cancer cells [25].

Several temporins with antimicrobial activities against Gram-positive bacteria (e.g., *S. aureus*, *B. megaterium*, and *Streptococcus pyogenes*), Gram-negative bacteria (e.g., *E. coli*, *P. aeruginosa*, *Aeromonas hydrophyla*), and fungi (*C. candida*) exhibited specific anticancer properties against cell lines like MCF-7 (human breast cancer), HeLa, and NCI-H460.

The AMP human cathelicidin LL-37 (hCAP18) that is derived from epithelial cells and leukocytes has two different mechanisms of action, depending on the type of cancer and also on the tissue origin of the tumor [26,27]. LL-37 can stimulate the growth, migration, and tumor formation in breast, prostate, and lung cancers, while in T-cell leukemia, colon, and gastric cancer, it inhibits metastasis [22,28].

Human intestinal defensin 5 (HD-5) has shown in vivo colon carcinogenesis activity without affecting the normal cells [29]. Another defensin, Laterosporulin 10 (LS10), displayed cytotoxicity against cancer cells (MCF-7, HEK293T, HT1080, HeLa, and H1299) without affecting the tested prostate epithelium cells (RWPE-1) [30].

The magainin II peptide demonstrated cytotoxic and antiproliferative effects against lung adenocarcinoma cells (A549) [31], bladder cancer cells (RT4, 647V, and 486P) [32], breast cancers cells (MDA-MB-231), and human mesothelioma (M14K) [33] while showing no impact on normal human fibroblasts (3T3) [32].

### 2.1. Buforin Peptides Identification

**Figure 1:**
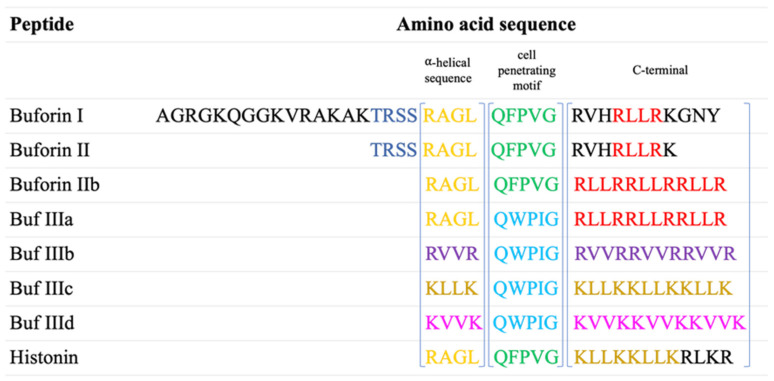
The amino acid sequence of buforins and their analogs.

Buforins are members of the non-lytic AMP family with the specific N-terminal section of histone H2A shared by all its members [34,35,36] (Figure 1). This portion of the protein is responsible for specifying the direct interaction with nucleic acids [35,37,38], inhibiting the cellular processes of the bacteria by binding to DNA and RNA [39]. They have significant antibacterial potential and promote inflammatory reactions, even though histone-derived peptides do not play a part in the replication process [34,35].

Kim et al. (2000) reported that the histone variant H2A was the precursor for a 39-amino acid sequence [40,41] named buforin I that was sampled and isolated for the first time from the gastric tissue of *Bufo bufo gargarizans*, and presented promising antibacterial effects against a broad spectrum of pathogens, both G+ bacteria (*B. subtilis*, *S. aureus*) and G- bacteria (*E. coli*, *S. typhimurium*) as well as fungi (*C. albicans*, *S. cerevisiae*) [35,40,42,43,44]. When buforin I was compared with other amphibian antimicrobial peptides (AMPs) like magainin 2, it demonstrated significantly stronger antimicrobial activities against microorganisms in vitro [40]. Magainin 2 is known to inhibit the growth of certain bacteria by preventing them from reproducing. This feature is achieved by binding to the bacterial membrane and disrupting its integrity. Magainin 2 also can inhibit the growth of certain fungi by preventing them from reproducing, similar to its activity on bacteria [45]. Buforin I is known to have bactericidal activity but also antiviral activity, which is achieved by binding to viral envelope proteins and disrupting the viral replication cycle [46].

Buforin II is a helix-hinge-helix residual 21 amino acid antimicrobial peptide derived from histones present in the gastric tissue of the Asian toad [34,38,40,43,47,48,49,50] or obtained with the help of Lys-C endoproteinase from buforin I, and usually contain some residues Thr^16^ to Lys^36^, presenting a higher antimicrobial activity than its parent peptide [40].

A derivative analog of buforin II named buforin IIb presents more substantial antibacterial and anticancer potentials [51,52,53,54] and includes at the C-terminus an α-helical sequence that exhibits in microorganisms a higher cytolytic activity than buforin II [38,50,55,56,57].

Derived from buforin II, Jang et al. designed several analogs named buforin III a-d. These derivatives maintained the crucial structural features and the general hydrophobicity necessary for exerting antimicrobial effects [58]. In obtaining buforin IIIa, the cell-penetrating motif was changed while keeping the rest of the structure identical to buforin IIb. For the following peptides, namely buforins III b, c, and d, the cell-penetrating motif from buforin IIIa was maintained, while the α-helical structure and C-terminal sequences were changed [58].

Furthermore, to obtain targeted medication delivery systems with reduced normal cell cytotoxicity and higher cancer cell affinity [59], several designers created cell-penetrating peptides like BR 1-3 which were synthesized and studied from the buforin IIb model [50,56,60].

### 2.2. Buforins’ Preparation and Characterization

Usually, AMPs have a low molecular weight and are composed of less than 50 amino acid bases, generally of a positive charge (+2 to +9) at pH 7 due to high lysine and arginine amounts [61]. More than 30% of their amino acids are hydrophobic [39,44,53,58].

Immunohistochemical (IHC) and biochemical analyses were used in determining that the mechanism of producing buforin I takes place within the gastric mucosal cells of the Asian toad after excessive production of histone H2A that surpasses the amount needed for DNA packaging and is further deposited in secretory granules [40,42,43]. Pepsin processes the extra histone H2A within the gastric lumen, resulting in the solid antimicrobial peptide buforin I, providing a protective layer to the stomach mucosal surface to which it adheres [40]. Within the stomach, the secreted HCl interacts with inactive pepsinogen, which is converted to active pepsin [40].

### 2.3. Buforins’ Mechanisms of Action

The molecular process of membrane penetration and the disruption of AMPs depends on various variables, such as the sequence of amino acids in the membrane, the amount of peptides, and the lipids in the membrane [39]. Almost all AMPs tend to be classified into two mechanistic pathways depending on their cell membrane interaction [61,62,63,64]. The first category focuses on cell wall disruption peptides that can distinguish between bacterial and human or host cells [43,65,66,67], and their cell wall penetrative properties include the following models: carpet, staves of a barrel, micellar aggregate, and toroidal pores (e.g., magainin-2, protegrin-1) [39,67]. Therefore, with the rise of AMPs in the volume outside the bacterial cells, different reactions eventually lead to the induction of apoptosis (inflammation or electrolyte disequilibrium) [62,68]. The second category of AMPs focuses on non-disruptive cell wall interactions (intracellular targets—altering protein synthesis, enzyme activity, binding, and interfering in DNA and RNA replication) [39,63,67]. Buforins are included in the latest category.

Even though the processes through which AMPs and members of the buforin family exhibit their action have not yet been fully explored and understood, models that have revealed intracellular and extracellular interference are generally recognized [61], and there is evidence that buforins can kill microorganisms by entering their cells and binding to nucleic acids [34,49,69,70]. Furthermore, members of this family can penetrate lipid bilayers thanks to the transient formation of peptide–lipid supramolecular complex pores [35].

Although there are similarities in the structure of buforin II and other AMPs (amphipathic α-helical peptides), their mechanism of action is different. Buforin II acts without bacterial cell lysis in vitro. It presents strong DNA and RNA affinity [34,38,40,69,71,72] and produces microbial cell aggregation [70]. Buforin II presents bactericidal effects for various G+ and G− microorganisms and fungi [73].

Several studies [49,74,75] compare the peptide–lipid interaction of buforin II to that of magainin 2 without producing significant lipid flip-flop or membrane permeabilization [40]. Other studies have observed that buforin II amide mimics the structure and composition of the Gram-negative bacterial cell membrane of *Escherichia coli*, adhering and generating peptide–DNA condensates inducing structural alterations within the bacterial cell [34,69,72,76,77], without disrupting the plasma membrane [40,43,53,58,70,78].

In rat models, it was tested individually or in association with antimicrobial drugs and had a high potential for treating *Acinetobacter baumannii* sepsis [34,70,78].

Buforin IIb is a histone-H2A-derived synthetic analog of buforin II [38,51] that contains an α-helical sequence (3xRLLR) [38] and is altered to improve its selectivity for cancer cells leading to cell death [52,61] without disrupting the normal cells [42,71]. Like buforin II, it interacts with the gangliosides in cancer cells, enters the cell without rupturing its membrane, and triggers mitochondrial apoptosis by activating caspase-9 [2,61] through mitochondria-dependent pathways [52]. Nonetheless, emerging evidence underscores the collaboration between the endoplasmic reticulum (ER) and mitochondria in signaling cell death. This investigation delves into the mechanism behind buforin-IIb-induced apoptosis in human cervical carcinoma HeLa cells, with a focus on ER stress-mediated mitochondrial membrane permeabilization [52].

Negatively charged elements present on cancer cells’ surfaces, like gangliosides, phosphatidylserine (PS), and heparan sulphate (HS) can serve as target molecules for interactions with peptides. The study published by Lee at. al in 2008 indicates that gangliosides on cancer cell surfaces act as particular binding sites for buforin IIb, enabling it to differentiate between cancer cells and normal cells [79].

Given the therapeutic potential and action mechanisms of buforin IIb against a broad spectrum of microorganisms, the possibility of developing resistance through target modification is unlikely [80]. However, even if buforin IIb has several remarkable characteristics of being a new antimicrobial or cancer treatment, the earlier analysis revealed that at higher amounts, it also attacked human cells [58].

To improve the therapeutic potential of the AMPs, analogs of buforin III, designated Buf III a-d, were synthesized from buforin IIb. These analogs were designed to retain key attributes such as hydrophobicity and certain structural elements crucial for maintaining antimicrobial efficacy. This strategic preservation ensures that the modified peptides exhibit potent antimicrobial activity while potentially offering an enhanced therapeutic index [58]. These analogs acted like their parent AMPs, easily penetrated bacterial cell membranes, and effectively were bound to DNA in vitro [58].

## 3. Anticancer Activity of Buforins

Cancer is a complex medical condition involving chronic low-level inflammation [81], and anticancer peptides (ACPs) are a category of AMPs capable of targeting cancer cell membranes. Although the inhibition or cancer-killing mechanisms are uncertain, there are similarities to those of antimicrobial activity, presenting both cell wall disruptive and non-disruptive mechanisms [62]. However, unlike healthy normal cell membranes, the surfaces of cancer cells have significant amounts of phosphatidylserine, which represents an important factor in cell selectivity and tumor specificity and delivers apoptosis [62,71].

Alongside cancer cell specificity, some ACPs like Dermaseptins B2 and B3 can restrain endothelial cell development and inhibit cancer cell proliferation [62]. Other ACPs lead to cancer cell apoptosis through DNA fragmentation. Both buforin II and buforin IIb present high anticancer activities and specificity through the cell surface gangliosides interaction [53,61] toward 60 cancer cell lines from the National Cancer Institute (USA) [50,52,70,79,82] like leukemia, CNS tumors, lung and renal cancers, prostate, melanoma, breast, and colon cancers [38,83], and display cytotoxic activity by disrupting mitochondrial functions causing apoptosis without affecting the cellular membrane [84].

### 3.1. Buforins Used for HeLa Cells

Recent studies explored the anticancer activity of buforins against HeLa cells, suggesting that these peptides have the potency to be delivery vectors and effective therapeutic agents [40,43,52]. Buforin I led to the growth inhibition and apoptosis of HeLa cells, presenting cytotoxic effects [35,42], and similar results were observed for buforin II [50,55,85]. Buforin IIb was determined to be highly selective toward HeLa cells by LC-MS/MS analysis, which confirmed its capability to enter cancer cells [86]. There are unknown mechanisms within the anticancer activity of buforins on HeLa cells, all demonstrating that buforin peptides accumulate at the cancer cell membranes without disrupting them [87] and inducing apoptosis through buforins’ intracellular accumulation [35,42]. Buforins also initiate cell death by binding to HeLa cell surface receptors, inhibiting cell proliferation and angiogenesis to slow tumor growth [50,55]. In vivo animal studies using models of HeLa cell xenografts have given important insights regarding buforins’ anticancer effects [59,79].

### 3.2. Buforins Used for Breast Cancer

Buforin IIb exhibited efficacy against breast cancer cells MX-1, MCF-7, and T47-D [79,82,88] with little or no effects on normal cells [71], where it hinders tumor growth in a mouse xenograft model by two mechanisms (anti-vasculogenic and anti-angiogenic). The glycosylation process in breast cancer cells facilitates their interaction with this antimicrobial peptide (AMP), exerting its anticancer effects [51,88]. A combination of doxorubicin and nisin had an anticancer effect against the MCF-7 cell line [89].

### 3.3. Buforins Used for Lung Cancer

In their study, Lee et al. (2008) [79] employed an NCI-H460 lung cancer cell line utilizing a transplantation technique to generate tumor xenografts. Their findings indicated that the administration of buforin IIb could effectively inhibit the progression of cancer xenografts in vivo (>5 mg/kg), suggesting that buforin IIb holds promise as a new therapeutic intervention for the management of cancer [38,61,79].

### 3.4. Buforins for Ovarian Cancer

For ovarian cancer cell lines (A2780 and A2780cisR) and normal non-cancerous human dermal fibroblasts (NHDF), in vitro cytotoxic analysis for testing cell viability and proliferation was performed using the microculture MTS assay. Buforin IIb presented similar cytotoxic results to *cis*-[Pt(NH_3_)_2_(malBuf_–2H_], a new Pt-buforin IIb conjugate and better than *cis*-Pt[(NH_3_)_2_(malonate)] [82], suggesting that peptides could have the potential of being a new therapeutic alternative while overcoming resistance [90].

### 3.5. Buforins for Prostate Cancer

Prostate cancer is one of the most frequent types of cancer present in male individuals. While the action mechanism of buforin IIb for this type of cancer is still not fully understood, it has been determined that it inhibits cell proliferation and induces apoptosis for PC-3 and Du-145 cancer cells in a dose-dependent manner (IC50 less than 8 µM) [51].

### 3.6. Buforins for Liver Cancer

Purified buforin IIb was tested on liver cancer cells HepG2 using different time frames and concentrations, analyzing cell viability and inhibitory effects. A concentration of 1.0 µM buforin IIb for 24 h presented the best results compared to control samples regarding cell proliferation and apoptosis. In vitro, almost 50% cell migration was observed, while in vivo experiments on HepG2 xenografts were performed on inoculated male mice. Tumor size (volume and weight) was inhibited, and cell apoptosis was observed [38].

## 4. Anticancer Activity of Buforin-Containing Bioconjugates

The search for effective and targeted anticancer therapies has led to exploring innovative strategies that leverage the unique properties of antimicrobial peptides. Bioconjugates incorporating antimicrobial peptides have emerged as promising novel and effective anticancer therapies [91].

As presented earlier, buforins, a family of small cationic peptides derived from histones, have demonstrated promising anticancer properties. Their ability to selectively target cancer cells while sparing normal cells makes them attractive candidates [66]. However, challenges such as low stability, limited bioavailability, and potential off-target effects have hindered their clinical translation. Researchers have explored the development of buforin-containing bioconjugates as innovative therapeutic strategies for cancer treatment to enhance their efficacy and specificity further. Harnessing the potential of buforins through bioconjugation offers a unique strategy to improve their specificity, bioavailability, and therapeutic efficacy in cancer treatment [55,82,92].

Bioconjugates are hybrid molecules that result from the covalent linkage of two or more distinct moieties, such as peptides, antibodies, or drugs, to create multifunctional entities [93]. This approach combines different therapeutic agents, enhancing their individual properties and enabling targeted drug delivery [94,95]. For buforins, several bioconjugation strategies have been explored, including peptide conjugation with nanoparticles, liposomes, and polymeric carriers. The carrier choice depends on the bioconjugate’s desired properties, including stability, release profile, and targeting ability. These approaches improve buforin stability and bioavailability, enabling controlled release and targeted delivery to cancer cells.

The bioconjugation of buforins with various entities, such as nanoparticles, antibodies, or targeting ligands, aims to improve their pharmacokinetics, biodistribution, cell-specific delivery, and targeting of cancer cells [96]. Strategies for buforin-bioconjugate design include chemical conjugation, genetic fusion, and supramolecular structures [2]. These approaches can modulate their physicochemical properties and tailor their anticancer activity [87]. Surface modification of nanoparticles or liposomes with targeting ligands, such as antibodies or peptides, enhances their specificity for cancer cell receptors, leading to increased cellular uptake [97,98].

Nanoparticle-based buforin bioconjugates, for example, can enable the slow and controlled release of the peptide, prolonging its presence within the tumor microenvironment and enhancing therapeutic efficacy. This targeted delivery minimizes off-target effects and improves the therapeutic index of buforin-containing bioconjugates. By incorporating buforins into carriers, researchers have achieved the controlled and sustained release of the peptides, leading to prolonged exposure to cancer cells. This sustained exposure amplifies the cytotoxic effects, resulting in improved efficacy against cancer cells.

The synthesis of a Pt(II)-peptide conjugate was reported by Parker et al. (2016) [82] using buforin IIb as a cell-penetrating guidance peptide and Pt(II) as a cancer cell cytotoxic load. The authors synthesized the D-stereoisomeric form of buforin IIb that was reacted with a malonate-type linker to obtain an O,O’-bidentate linker that was further complexed with *cis*-[Pt(NH_3_)_2_(H_2_O)_2_]^2+^ to produce *cis*-[Pt(NH_3_)_2_(malBuf_–2H_)]. The amino acid sequence side chains from buforin IIb are compatible and non-reactive with Pt, resulting in a Pt-buforin IIb conjugate more cytotoxic and selective to the ovarian cancer cell line (A2780cisR) than the parental AMP buforin IIb. Additionally, combining buforins with other anticancer agents in bioconjugates exhibited synergistic effects, leading to greater effectiveness in inhibiting tumor growth and inducing apoptosis.

Libardo et al. (2015) [99] obtained an *sh*-buforin (RAGLQFPVGRVHRLLRK-NH_2_) conjugate containing an amino-terminal Cu^2+^ and Ni^2+^ binding unit (ATCUN): ATCUN-AMP. The ATCUN motif influenced the nuclease activity and made these peptide conjugates more active and stable than the parental AMP [91]. The antibacterial potency was influenced by the stereochemistry of the derivatives containing L- and D-amino acids in the AMP sequence; it was higher for ATCUN-D-*sh*-buforin than ATCUN-L-*sh*-buforin.

In their study, Cuellar et al. (2018) [100] immobilized buforin II on magnetite nanoparticles. The results indicate that membrane translocation succeeded without promoting disruption and affecting cell viability in the mammalian and bacterial cells investigated. However, antimicrobial activity was affected; buforin-II–magnetite conjugates presented no antimicrobial activity compared to buforin II.

Another study on magnetite nanoparticles and buforin II conjugates was performed by Perez et al. (2019) [92]. For better molecular flexibility, buforin II was immobilized on polyetheramine-modified magnetite to increase the penetration properties in diverse cell-lines, with high biocompatibility but with a decrease in antimicrobial effects compared to the parent peptide.

A specific anticancer effect of a buforin IIb and anionic peptide (a derived magainin sequence) conjugate, bonded via a matrix metalloproteinase (MMP) cleavable linker, was obtained and investigated by Jang et al. (2011) [55]. The resulting buforin conjugate presented better anticancer effects than buforin alone in the pathological-producing MMP cells.

One of the major challenges in cancer treatment is the development of drug resistance in cancer cells. Bioconjugates offer a potential solution by bypassing drug efflux pumps and other resistance mechanisms [94,95].

Combination therapies involving buforin-containing bioconjugates with chemotherapeutic drugs or other AMPs have shown promising results in preclinical studies. The synergistic effects of these combinations result in enhanced cytotoxicity and reduced drug resistance in cancer cells [91]. Bioconjugates can facilitate the co-delivery of multiple therapeutic agents, maximizing their combined anticancer potential. While promising, translating buforin-containing bioconjugates from preclinical studies to clinical applications requires rigorous evaluation. In vivo studies have provided valuable insights into their pharmacokinetics, toxicity profiles, and antitumor effects [82]. Future clinical trials will be crucial in assessing the safety and efficacy in human subjects. Further research is needed to optimize bioconjugation strategies, assess long-term safety, and conduct clinical trials to evaluate their effectiveness and tolerability in cancer patients.

Buforin-containing bioconjugates represent a novel and promising approach in cancer therapy, combining the unique anticancer properties of buforins with the benefits of bioconjugation strategies. Bioconjugation enhances the anticancer potential by improving specificity, cellular uptake, and therapeutic efficacy while mitigating off-target effects and drug resistance. The improved targeting, sustained activity, and synergistic effects with other therapeutic agents make these bioconjugates attractive candidates for personalized and targeted cancer treatment. As research progresses, the development of buforin-containing bioconjugates holds great potential in improving cancer patient outcomes and advancing the field of anticancer therapeutics. This strategy holds promise for overcoming the limitations of free buforins and offers a novel and exciting direction in this field. Continued research and development in this area may eventually lead to the clinical use of buforin bioconjugates as a potent and selective therapy against cancer.

## 5. Conclusions and Perspectives

Non-communicable illnesses, especially cancer, continue to pose a significant global public health challenge. Despite decades of research and therapeutic approaches, cancer remains a major cause of morbidity and mortality worldwide. Chemotherapy, a commonly used treatment, has several drawbacks, including non-selective targeting and drug resistance. To address these issues, scientists in the pharmaceutical industry are exploring various approaches, including nanocarriers and antibody–drug conjugates, to improve cancer treatment efficacy and reduce side effects.

Among the potential solutions, using selenium-conjugated peptides (STPs) and marine peptides show promise as alternative cancer treatments. The search for novel antimicrobial medications has become increasingly urgent due to the growing problem of microbial resistance to conventional antibiotics. AMPs from various sources, such as plants, insects, amphibians, and animals, have demonstrated strong antimicrobial activity against bacteria, fungi, viruses, and parasites. These AMPs offer potential as novel therapeutics to combat infectious diseases and cancer.

The discovery and development of targeted therapies, nanotechnology-based approaches, and natural peptides like AMPs provide hope for advancing cancer treatment and antimicrobial strategies. As research in these areas continues, the medical community can look forward to improved treatments that enhance patient outcomes and quality of life. However, further research and clinical trials are needed to fully understand and harness the potential of these novel therapeutic strategies.

Antimicrobial peptides (AMPs), such as the buforin family, have emerged as a promising source of new antibiotics. Buforins exhibit potent antimicrobial and anticancer properties, by disrupting bacterial cell membranes or interfering with intracellular processes. They offer potential advantages over conventional antibiotics, including fast action, reduced resistance buildup, and environmental friendliness.

The practical application of buforins as therapeutic agents faces challenges such as high production costs, limited bioavailability, and potential toxicity to mammalian cells. To address these limitations and enhance the therapeutic potential of buforins, researchers have explored the development of buforin-containing bioconjugates. Bioconjugation strategies involving the covalent linkage of buforins with nanoparticles, liposomes, or targeting ligands aim to improve their stability, bioavailability, and specificity for cancer cells. These bioconjugates offer controlled release and targeted delivery, minimizing off-target effects and potentially overcoming drug resistance in cancer cells.

While promising, translating buforin-containing bioconjugates from preclinical studies to clinical applications requires further evaluation and optimization. In vivo studies using animal models have provided valuable insights, and future clinical trials will be essential to assess their safety and efficacy in human subjects.

Developing buforin-containing bioconjugates represents a significant step forward in the quest for effective and targeted antimicrobial and anticancer therapies. Continued research and clinical investigations will determine their potential as valuable additions to the arsenal of modern medicine in the fight against infectious diseases and cancer.

## Data Availability

No data was used for the research described in the article.
